# Clinical Society

**Published:** 1897-03-06

**Authors:** 


### Medical Progress and Hospital Clinics.

[*The Editor will be glad to receive offers of co-operation and contributions from members of the profession. All letters should be addressed to* The Editor, at the Office, 28 & 29, Southampton Street, Strand, London, W.C.]

### CLINICAL SOCIETY.

A curious and interesting case of "dual consciousness" was shown by Dr. Albert Wilson at the last meeting. The patient was a girl, aged 14½ years, and the illness began with influenza, followed by a relapse and meningitis, in April, 1895. During the second week of her illness the sight was affected, and she had to be kept in the dark. At this period also there was great sensitiveness to sounds. In the fourth week mania developed, with delirium and intense fear. She had delusions and hallucinations, calling people around her snakes, and at this time she manifested great strength. During the third week, whilst her sight was affected, she recognised her parents by touch and not by sight. Later on she developed muscular twitchings, which went on to complete opisthotonos. As the mania passed off her mental condition was found to have changed, and among other things she gave fresh names to those around, calling her father "Tom," her mother "Mary Ann," one doctor "Jim," and another "Sam." Physically she improved; the sight got much better, but she remained paralysed in the legs. After the fifth week the curious condition of dual personality began to show itself. While sitting in bed playing with her dolls she would say, "It is coming," and push all the toys to one side, then there would be a shaking of the legs; then she would turn a summersault and sit up in a new personality, generally calling out "Holloa," as if she had just arrived. In this abnormal condition she called those around by the nick-names she had given them at the time of the mania, which suggested some connection between this abnormal state and the mania which had preceded it. During this abnormal or "B" stage, she talked baby talk, clipping words and not recognising the names of things. This stage lasted from ten to fifty minutes, and when she recovered she was dazed for two or three minutes, when she would begin again playing with her dolls at the point where she had left off. Thus as Dr. Wilson said, "The 'A' or normal stage is continuous, and the 'B' or abnormal stage is also continuous; but 'A' knows nothing of 'B,' and 'B' knows nothing of 'A.'" About this time attacks of catalepsy began to come on, sometimes affecting one limb only, sometimes the whole of the body, and by degrees the abnormal state came on more frequently, or rather lasted longer, so that after two or three months she was almost as frequently abnormal as normal. Up to July, 1895, she was still powerless in the legs, and had to be taken out in a bath-chair; but suddenly, on July 20th, her power of walking returned perfectly. Since this period several variations have occurred in the 'B' or abnormal state, and each of these sub-states seem at first to be absolutely distinct from those that have gone before; but as time passes on there develops a hazy knowledge in one sub-state of 'B' of what has occurred in other sub-states of 'B,' and also in these 'B' states she has some slight idea of what occurs in her normal condition; but while she is in the 'A' or normal state she knows absolutely nothing of what occurs in the abnormal conditions. Dr. Wilson had recorded many of the very peculiar actions and mental conditions which occurred in the various phases of the abnormal state, among which it is worth mentioning that while in this condition she used to steal things; but one day, outside a shop, she took an apple—on seeing a policeman, however, she put it back again. After severe attacks of catalepsy she became deaf and dumb for some hours, and this condition would pass off suddenly. She menstruated for the first time early in December, 1896, but no change occurred in her mental state. In January, 1897, she became quite blind and imbecile, and had to be guided by sound and touch. But on January 19th she menstruated for the second time, and after that improved much in health, so that she could walk, and could see things at a distance of about three inches. During the abnormal state there was a certain protrusion of the eyeballs which, however, disappeared when she returned to the normal mental state. Mr. Tweedy had examined her eyes, and had reported that the discs and fundus were healthy.

We have not space to describe the many peculiarities which had been noted by Dr. Wilson. One interesting point, however, should be mentioned, that although in the normal she was not known to have any knowledge of drawing, she made some rather clever memory sketches while in the "B" state, clearly from remembered fashion-plates, and some of these she drew "when stone-blind and when a book was placed between her eyes and the paper."

It is worth noting that this case did not seem to be one of double consciousness in the ordinary acceptation of the term. There was an alternation between an ordinary state and a considerable variety of abnormal conditions, but there were not, as has so often been described, two separate mental states each continuous in itself, yet each independent of the other. The case is none the less interesting on this account; perhaps, it is even more so, as tending by its superficial resemblance to cases of dual consciousness on the one hand, and by its clear connection with so-called hystero-epilepsy on the other, to show the relation of these two conditions.

By several of those who examined the case its strong association with hysteria and other allied conditions was insisted on, and it was urged that isolation from her home surroundings would be a very necessary part of the treatment.

